# Neural evidence for a bilingual advantage in conflict monitoring among Dai bilinguals

**DOI:** 10.1038/s41598-025-31944-9

**Published:** 2025-12-15

**Authors:** Rui Yu, Jiemin Zhang, Fuhua Yang, Jiaci Lin

**Affiliations:** 1 School of Nursing, Yunnan University of Chinese Medicine, Kunming, 650500 China; 2 Faculty of Education, Yunnan Normal University, Kunming, 650500 China; 3 School of Information, Yunnan University of Chinese Medicine, Kunming, 650500 China; 4 School of Social and Behavioral Science, Nanjing University, Nanjing, 210023 China

**Keywords:** Bilingual advantage, Conflict monitoring, Inhibitory control, Conflict effect, Congruency sequence effect, Neuroscience, Psychology, Psychology

## Abstract

Research on the bilingual advantage in cognitive control has yielded mixed results, particularly across diverse populations. This study examined whether Dai bilinguals in China demonstrate enhanced cognitive control compared to monolinguals. Participants completed a classic Eriksen Flanker task while both behavioral responses and EEG data were recorded. Analyses focused on reaction times, conflict effects, congruency sequence effects, and the ERP components N2 (associated with conflict monitoring) and P3 (associated with attentional allocation). Although no significant group differences emerged in behavioral performance, bilinguals showed reduced N2 and increased P3 amplitudes relative to monolinguals, indicating group differences in neural correlates of conflict monitoring. No differences were observed in conflict or congruency sequence effects between the groups. These findings suggest a bilingual experience may be associated with differences in the neural dynamics of conflict monitoring, even in the absence of behavioral differences. The effects were not lateralized. This highlights the value of combining behavioral and ERP measures to investigate bilingual cognitive processing in non-Indo-European language contexts.

## Introduction

With the advancement of human civilization and increasing globalization, an ever-growing number of individuals have become bilingual. Throughout much of the twentieth century, however, bilingualism was discouraged by educators who believed that managing two languages hindered learning abilities and led to greater academic and intellectual deficits compared to monolingualism ^1^. This view began to change with the study by Peal and Lambert, which demonstrated that bilingual individuals performed significantly better on both verbal and nonverbal measures of intelligence^2^.

In the past two decades, there has been a surge of research investigating whether bilingualism confers a cognitive advantage. This interest has led to a proliferation of studies in psychology and education focusing on the relationship between bilingualism and cognitive control functions. Many of these studies have employed cognitive control tasks involving congruent (C) and incongruent (I) conditions, such as the Flanker, Simon, Stroop, and Attention Network Task (ANT). However, findings in this area remain inconsistent. Some studies report superior cognitive performance among bilinguals, supporting the bilingual advantage hypothesis^3–5^ or that the advantages of bilingualism outweigh its disadvantages^6^, while others have found no such evidence^7–9^. Therefore, further empirical evidence and theoretical discussion are required to determine whether bilingual experience truly confers cognitive advantages.

The fundamental assumption underlying the bilingual advantage hypothesis is that bilinguals activate both languages simultaneously. When confronted with different communicative demands, they must resolve interlanguage conflict through a control mechanism that selects between the two linguistic systems. A broader theoretical framework, the adaptive control hypothesis^10^, emphasizes that language control processes adapt to the recurrent demands of different interactional contexts. While the present study does not directly examine interactional context, this framework provides a useful background by suggesting that bilingual cognitive control may be supported by different mechanisms under different demands. Within this broader perspective, two primary explanations have been proposed.

One prominent account is the Inhibitory Control Theory^11^, which posits that bilinguals must suppress activation of the non-target language during use of the target language. This constant practice is thought to enhance their inhibitory control compared to monolinguals. In empirical research, inhibitory control is typically measured by the difference in mean reaction times between incongruent and congruent trial conditions-known as Conflict Effects (CE)^12^. When present, a bilingual advantage in inhibitory control is reflected in smaller Conflict Effects for bilinguals compared to monolinguals^13,14^.

Another explanation is the Conflict Monitoring Theory ^15^. According to this theory, the bilingual advantage may not stem from an enhanced ability to suppress interfering information, but rather from superior monitoring abilities in rapidly changing and complex tasks. This advantage likely arises from the need for bilinguals to consistently attend to and select the appropriate language for each communicative context. As a result, bilinguals may develop an improved capacity to monitor complex environments where both relevant and irrelevant stimuli are present. The theory also posits that specific brain regions, particularly the anterior cingulate cortex (ACC), are responsible for conflict monitoring. Two indicators are commonly used to measure monitoring function. The first includes overall reaction time as well as electrophysiological indices such as the N2 and P3 amplitudes. The N2 amplitude is generally taken to reflect early detection of cognitive conflict, while the P3 amplitude is linked to attentional engagement and stimulus evaluation^3,14,16^. Hilchey and Klein found that bilinguals exhibited shorter reaction times than monolinguals in both congruent and incongruent tasks, which was interpreted as reflecting bilinguals’ enhanced monitoring abilities^17^. The second indicator is the Congruency Sequence Effect (CSE), also known as the Gratton effect or conflict adaptation effect^18^. According to Conflict Monitoring Theory, incongruent trials trigger greater ACC activation compared to congruent trials, which in turn increases attention to the conflict and reduces interference in subsequent trials. This phenomenon is reflected by shorter reaction times in inconsistent trials followed by inconsistent trials (iI) compared to inconsistent trials followed by congruent trials (cI), i.e., iI < cI; and in consistent trials followed by consistent trials (cC) compared to inconsistent trials followed by consistent trials (iC), i.e., cC < iC. Both effects may co-occur, as indicated by the finding that the conflict effect in consistent trials followed by consistent trials is greater than that in inconsistent trials followed by consistent trials, i.e., cI—cC > iI—iC. The CSE size is calculated as the difference between the conflict effects after consistent and inconsistent trials, i.e., CSE = (cI—cC)—(iI—iC)^19,20^. A larger CSE suggests that prior trial conditions have a greater influence on current performance, with slower attentional separation from previous trials^21^. The more effective conflict monitoring system of bilinguals may provide them with an advantage in conflict adaptation, as evidenced by a lower CSE ^17^.

In addition to the theoretical accounts of bilingual cognitive control, electrophysiological studies have provided valuable insights into the underlying neural mechanisms. Two ERP components have been particularly informative in this regard: the N2 and P3. The N2, typically observed with a fronto-central distribution ^22^, is associated with conflict monitoring and early detection of cognitive conflict reflecting activity in the anterior cingulate cortex (ACC) ^14,23^. The P3, primarily recorded in central-parietal regions ^24,25^, is linked to attentional allocation and stimulus evaluation ^26^. Previous studies have suggested that bilinguals may exhibit reduced N2 amplitudes and enhanced P3 amplitudes compared to monolinguals in some cognitive control tasks, which could reflect more efficient conflict monitoring and greater attentional engagement ^3,14,27^. These findings indicate that ERP measures can capture subtle neural differences between bilinguals and monolinguals that may not be evident in behavioral performance alone.

Existing studies have predominantly focused on behavioral experiments, mainly examining measures such as reaction time and accuracy^28^. Compared to behavioral experiments alone, cognitive neuroscience methods are more sensitive and crucial for elucidating the complex relationship between bilingualism and cognitive control ^27,29^. Although an increasing number of recent studies have employed cognitive neuroscience approaches to investigate bilingual advantages, research that, to our knowledge, systematically distinguishes and simultaneously analyzes both conflict monitoring and inhibitory control mechanisms remains limited ^30^. In particular, there is a lack of studies that comprehensively examine all three indicators related to these two potential mechanisms. To address this gap, the present study employed both behavioral and electrophysiological indices to differentiate these cognitive mechanisms. Inhibitory control was measured using the Conflict Effect (CE), derived from both reaction times and ERP amplitudes. Conflict monitoring was assessed using overall reaction times, N2 and P3 amplitudes, and the Congruency Sequence Effect (CSE), calculated for both behavioral and ERP data. This combination enables a reasonable operationalization of each mechanism and facilitates interpretation of observed ERP and behavioral effects within the theoretical framework.

Globally, bilingualism research has predominantly focused on bilinguals with English as a second language, with minimal attention to ethnic minority groups. In recent years, the promotion of China’s national lingua franca in ethnic regions has accelerated, leading to a significant increase in the number of Chinese-ethnic bilinguals among ethnic minorities. However, few empirical studies have investigated the impact of bilingual experiences on the cognitive development of ethnic minority groups. The Dai people, a unique ethnic minority in Yunnan, China, represent the most populous cross-border ethnic group in the country. The Dai language belongs to the Zhuang-Dong branch of the Sino-Tibetan language family, a distinct branch from Chinese, and is spoken by approximately 68 million people worldwide. Dai bilinguals thus constitute a relevant representative sample of ethnic minority bilinguals.

Thus, the present study aims to examine Dai college students as representatives of ethnic bilinguals, using the event-related potential (ERP) technique and the classical Flanker paradigm to explore the following research questions: (1) Does a bilingual advantage exist in a cognitive task involving conflicting information? (2) If a bilingual advantage exists, is it driven by inhibitory control or conflict monitoring? (3) If a conflict-monitoring advantage exists, does it arise from monitoring itself, or from the adaptation of monitoring to conflict? (4) Is the bilingual advantage associated with greater left-hemisphere neural activation, reflecting potential effects of language experience on left-hemisphere activity?

## Methods

### Participants and procedure

A total of 48 participants participated in the experiment. One participant was excluded due to low accuracy in the behavioral task (below 75%), and two participants were excluded due to excessive EEG artifacts. The final valid sample consisted of 45 participants. All participants were Dai college students from Xishuangbanna Dai Autonomous Prefecture, Dehong Dai Jingpo Autonomous Prefecture, Gengma Dai Wa Autonomous County, Menglian Dai Lahu Wa Autonomous County, and Lincang, Yunnan Province, China, aged between 18 and 22 years. The monolingual group included 23 participants (16 females and 7 males, mean age = 19.52 ± 0.99), while the bilingual group included 22 participants (15 females and 7 males, mean age = 19.73 ± 1.12). The two groups did not differ significantly in terms of age (*t*(43) = -0.652, *p* = 0.518) or gender distribution (χ2(1) = 0.010, *p* = 0.920).

Participants in the monolingual group reported proficiency only in Chinese, with no listening skills in Dai. In contrast, participants in the bilingual group reported Dai as their first language and Chinese as their second language, with proficiency in listening to both languages. All bilingual participants were early acquirers of their second language, with the mean age of second language acquisition being 4.59 years (SD = 1.764, range 2–8 years). The bilingual group had a significantly higher age of Chinese language acquisition than the monolingual group (*t*(43) = 7.113, *p* = 0.000).

Language proficiency was assessed using a five-point self-assessment scale, where 1 indicated no proficiency and 5 indicated full proficiency. Both groups demonstrated high proficiency in Chinese (monolinguals: M = 4.87; bilinguals: M = 4.73), while the bilingual group also reported high proficiency in Dai (M = 4.08), in contrast to the monolingual group (M = 1.09). Statistical analysis further showed no significant difference in Chinese proficiency between the two groups (*t*(43) = 1.185, *p* = 0.243), but the bilingual group exhibited significantly higher Dai proficiency than the monolingual group (*t*(43) = 14.864, *p* = 0.000). This indicates that the bilingual participants were proficient users of both languages, with mean scores above 4 out of 5 in each.

Since family socioeconomic status (SES) has been shown to influence cognitive performance^31,32^, the study also considered participants’ SES. Family SES was assessed using three indicators: parents’ education level, parents’ occupation, and family income. Given that many college students are unaware of their family income, this study focused on parents’ education level (1. Primary school and below; 2. Primary school; 3. High school or junior college; 4. College; and 5. Graduate school and above) and occupation. The standardized scores for parents’ education level and occupation were summed to represent the participants’ family SES. No significant difference in SES scores was found between the monolingual and bilingual groups (*t*(43) = -0.865, *p* = 0.392). Relevant background information is summarized in Table 1.

**Table 1:** Participant background information.

	Monolingual	Bilingual	*t*
N	23	22	–
Age	19.52 (0.99)	19.73 (1.12)	− 0.652
Age of acquisition of Dai language	–	1.59 (0.854)	–
Age of acquisition of Chinese language	1.65 (0.885)	4.59 (1.764)	7.113 ***
Proficiency in Dai language	1.09 (0.294)	4.08 (0.900)	14.864 ***
Proficiency in Chinese	4.87 (0.344)	4.73 (0.456)	1.185
SES	0.422 (3.13)	-0.442 (3.56)	− 0.865

***Represents *p* < .001.

All participants were right-handed and had normal or corrected-to-normal vision. The study complied with relevant ethical standards and was approved by the Ethics Committee of Yunnan University of Chinese Medicine. After providing informed consent, participants completed the Flanker task following the collection of background information. The entire experiment lasted approximately 40 min, and participants received a reward upon completion.

### Flanker task

The experimental program was developed using E-Prime 2.0 software, and stimuli were presented at the center of a 19-inch computer screen positioned approximately 50 cm from the participant. The screen refresh rate was 75 Hz. The task was a classic arrow-based Flanker paradigm, using five arrows (< or >) displayed in 18-point Verdana font. There were four stimulus types: the congruent condition (C), in which all arrows pointed in the same direction (e.g., >  >  >  >  > or <  <  <  < <), and the incongruent condition (I), in which the central arrow pointed in the opposite direction from the flanking arrows (e.g., >  >  <  >  > or <  <  >  < <). Each trial began with a variable inter-trial interval (ITI, 300–500 ms), randomly assigned across trials, followed by the stimulus presentation. Participants were instructed to ignore the flanking arrows and respond only to the direction of the central arrow. If the central arrow pointed left ( <), participants pressed the “S” key with their left index finger; if it pointed right ( >), they pressed the “K” key with their right index finger. Responses were required within 2000 ms, after which the program automatically proceeded to the next stimulus.

The experiment consisted of nine blocks: one practice block and eight formal blocks. The practice block included 16 trials-8 congruent and 8 incongruent. Participants proceeded to the formal experiment only after achieving an accuracy rate of at least 85% in the practice block.

In the formal phase, each of the four stimulus types was presented 64 times, resulting in a total of 256 trials. Because the congruency sequence effect (CSE) involves the influence of the previous trial on the current one, trials were pseudo-randomized to ensure a balanced number of transitions between trial types and to precisely define trial categories. Excluding the first trial of each block (which had no preceding trial), the remaining 248 trials included 62 trials in which both the previous and current trials were congruent (cC), 62 trials in which the previous trial was congruent and the current trial was incongruent (cI), 63 trials in which the previous trial was incongruent and the current trial was congruent (iC), and 61 trials in which both the previous and current trials were incongruent (iI).

**Fig. 1:**
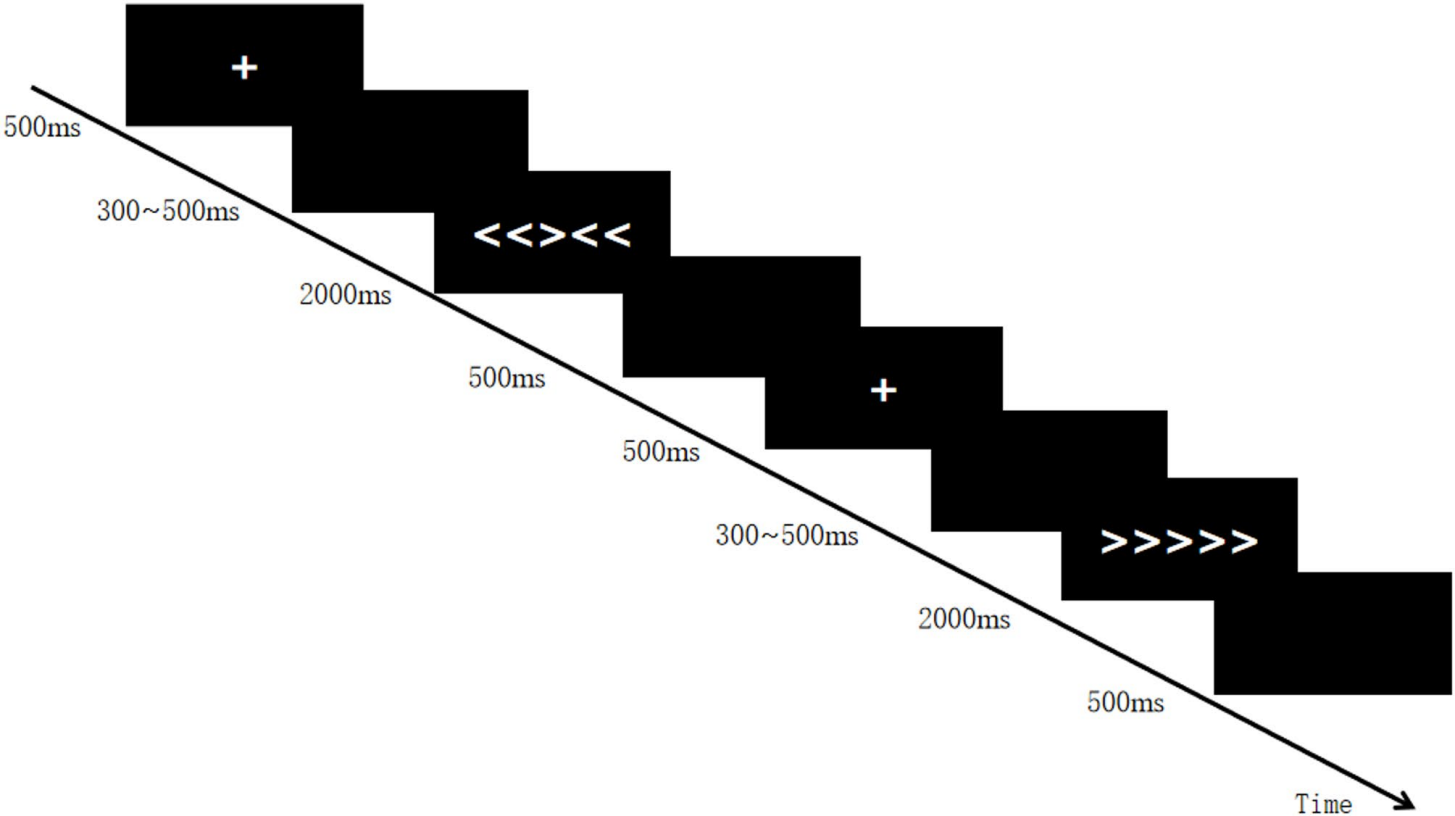
Example of experimental flow.

All 256 trials were presented in a predetermined order and divided into eight blocks of 32 trials each. After each block, participants were given a rest period, the duration of which they could control themselves. The detailed experimental procedure is illustrated in Fig. 1.

The example in the procedure illustrates a case in which the first stimulus is an incongruent type (I) and the second stimulus is a congruent type (C). Considering the sequential relationship between trials, the second stimulus is thus categorized as an iC trial.

### Data acquisition and preprocessing

Behavioral data, including participants’ keypress reaction times and error rates, were recorded using E-Prime 2.0. EEG data were recorded using a 64-channel EEG amplifier system (Brain Products GmbH, Germany), with electrodes positioned according to the international 10–20 system. The online reference was set at FCz, and the ground electrode was located at the midpoint between FPz and Fz. Horizontal electrooculogram (HEOG) signals were recorded using an electrode placed at the outer canthus of the right eye. The data were sampled at 500 Hz and recorded with an online band-pass filter ranging from 0.01 to 70 Hz. Scalp electrode impedances were maintained below 5 kΩ throughout the recording.

Offline EEG processing was performed using EEGLAB 13.0 running on MATLAB R2013a. Continuous EEG data were first re-referenced to the average of the left and right mastoid electrodes. Independent Component Analysis (ICA) was applied to the continuous data to identify and remove components associated with ocular artifacts, including eye blinks. Subsequently, the data were filtered using a 0.01–30 Hz finite impulse response (FIR) band-pass filter with a transition bandwidth of approximately 0.5 Hz and a roll-off rate of ~ 12 dB/octave. Epochs were extracted from − 200 ms to 800 ms relative to stimulus onset, and baseline correction was performed using the pre-stimulus interval (-200 to 0 ms). Epochs were excluded from averaging if the amplitude exceeded ± 80 μV in any EEG channel.

The ERP components of interest were N2 and P3^21^. The N2, a negative deflection associated with early conflict detection^14,23^, typically exhibits a fronto-central distribution ^22^. To examine hemispheric differences, seven frontal electrode sites were selected: left frontal (F1, F3, F5), frontal midline (Fz), and right frontal (F2, F4, F6). The P3 component, a positive wave linked to attentional resource allocation^26^, is primarily observed in the central-parietal region^24,25^. For P3 analysis, seven central-parietal electrodes were selected: left central-parietal (CP1, CP3, CP5), central-parietal midline (CPz), and right central-parietal (CP2, CP4, CP6). To avoid bias in time-window selection, time windows for the N2 and P3 components were determined based on the grand-averaged waveforms across all participants and conditions at the respective frontal and centro-parietal electrode sites^33^. The time windows were defined around clear peaks of these waveforms, resulting in windows of 280–360 ms for N2 and 360–500 ms for P3.

Behavioral reaction times and EEG data analyses included only correct trials, excluding practice trials, the first trial of each block, trials with reaction times below 200 ms or above 1000 ms, trials outside 2.5 standard deviations from the mean reaction time, and trials immediately following errors. After applying the exclusion criteria, the acceptance rates were 85.45% for the monolingual group and 86.83% for the bilingual group.

### Statistical analyses

Behavioral and EEG data were analyzed using SPSS 22.0. The Greenhouse–Geisser correction was applied when the sphericity assumption was violated, and multiple comparisons were adjusted using the Bonferroni correction. Statistical significance was set at α = 0.05.

In addition to the omnibus ANOVAs, two widely used indices of conflict processing—conflict effect (CE = incongruent − congruent) and congruency sequence effect (CSE = (cI − cC) − (iI − iC))—were calculated to provide a more direct and interpretable assessment of immediate conflict costs and dynamic conflict adaptation. This approach complements the omnibus ANOVA results and facilitates comparisons with previous studies. The same analytical rationale was applied to both behavioral and ERP data. Conflict Effect (CE) was used to index inhibitory control. Overall reaction times, N2/P3 amplitudes, and the Congruency Sequence Effect (CSE) were used to index conflict monitoring, allowing for a precise operationalization of these two cognitive mechanisms across behavioral and ERP measures.

## Results

### Behavioral experiments-overall response time analysis

A 2 (Group: monolingual, bilingual) × 2 (Previous Trial Consistency: congruent [c], incongruent [i]) × 2 (Current Trial Consistency: congruent [C], incongruent [I]) three-way repeated measures ANOVA was conducted with response time as the dependent variable. Group served as a between-subjects factor, while previous and current trial consistencies were within-subjects factors. Accuracy rates across all conditions exceeded 95%, so no further analyses of accuracy were performed.

**Fig. 2:**
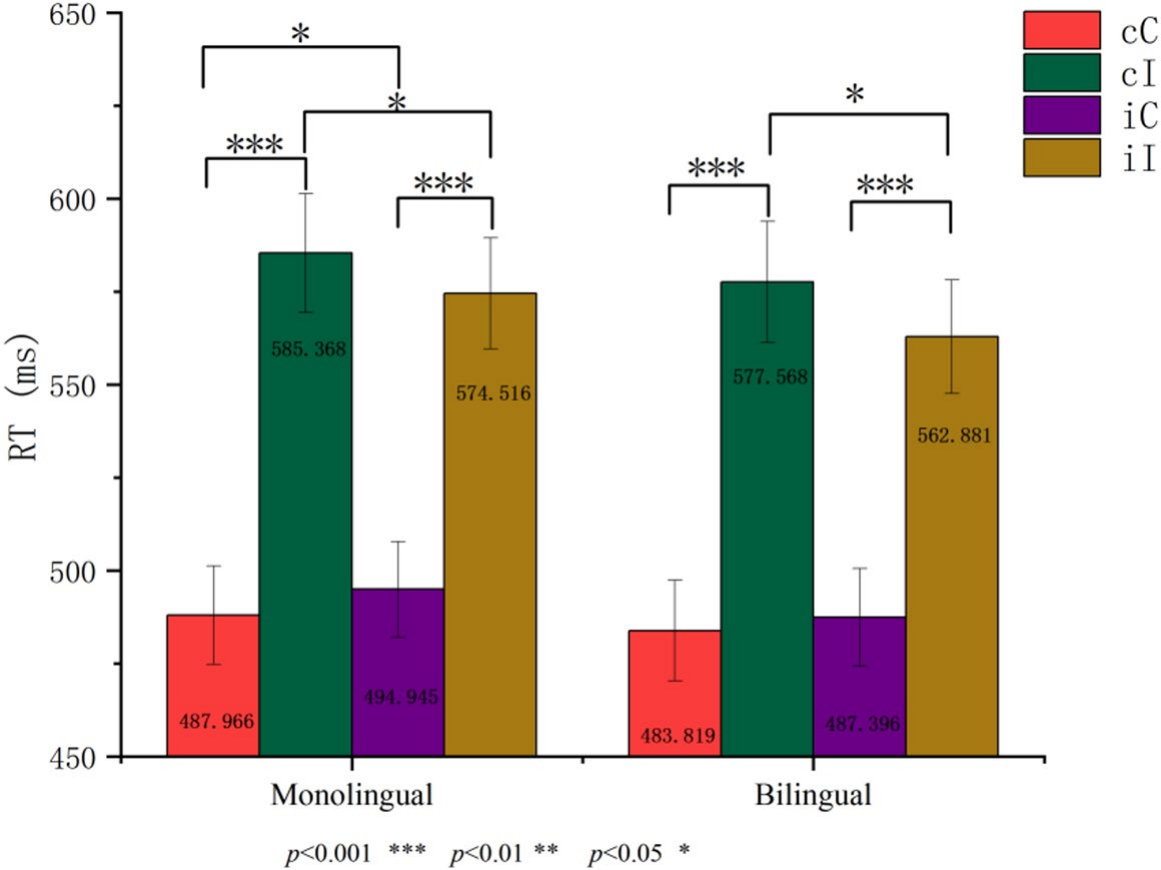
Overall reaction time results.

Analysis of reaction times (see Fig. 2) revealed no significant main effect of Group,* F*(1, 43) = 0.154, *p* = 0.697, but a significant main effect of Current Trial Consistency, *F*(1, 43) = 493.237, *p* < 0.001, η_p_^2^ = 0.920, with longer reaction times observed in incongruent compared to congruent trials. Neither the Group × Previous Trial Consistency interaction, the Group × Current Trial Consistency interaction, nor the three-way interaction among Group, Previous Trial Consistency, and Current Trial Consistency reached significance (all *F*s < 1).

A significant interaction between Previous Trial Consistency and Current Trial Consistency was observed, *F*(1, 43) = 16.019, *p* < 0.001, η_p_^2^ = 0.271. As expected, incongruent trials elicited longer reaction times than congruent trials (*p*s < 0.001). More importantly, when the current trial was congruent, reaction times were longer if the previous trial was incongruent compared to congruent (iC > cC, *p* = 0.020). Conversely, when the current trial was incongruent, reaction times were longer if the previous trial was congruent rather than incongruent (cI > iI, *p* = 0.001).

### Behavioral experiments- analysis of conflict effects

A 2 (Group: monolingual, bilingual) × 2 (Conflict Effect Type: post-consistent trial, post-inconsistent trial) repeated measures ANOVA on the conflict effect magnitude in response time (CE = I − C) revealed no significant main effect of Group, *F*(1, 43) = 0.246, *p* = 0.622. However, the main effect of Conflict Effect Type was significant, *F*(1, 43) = 16.019, *p* < 0.001, η_p_^2^ = 0.271, indicating that the conflict effect following consistent trials was significantly larger than that following inconsistent trials. The interaction between Group and Conflict Effect Type was not significant, *F*s < 1.

### Behavioral experiments- analysis of congruency sequence effect

**Fig. 3:**
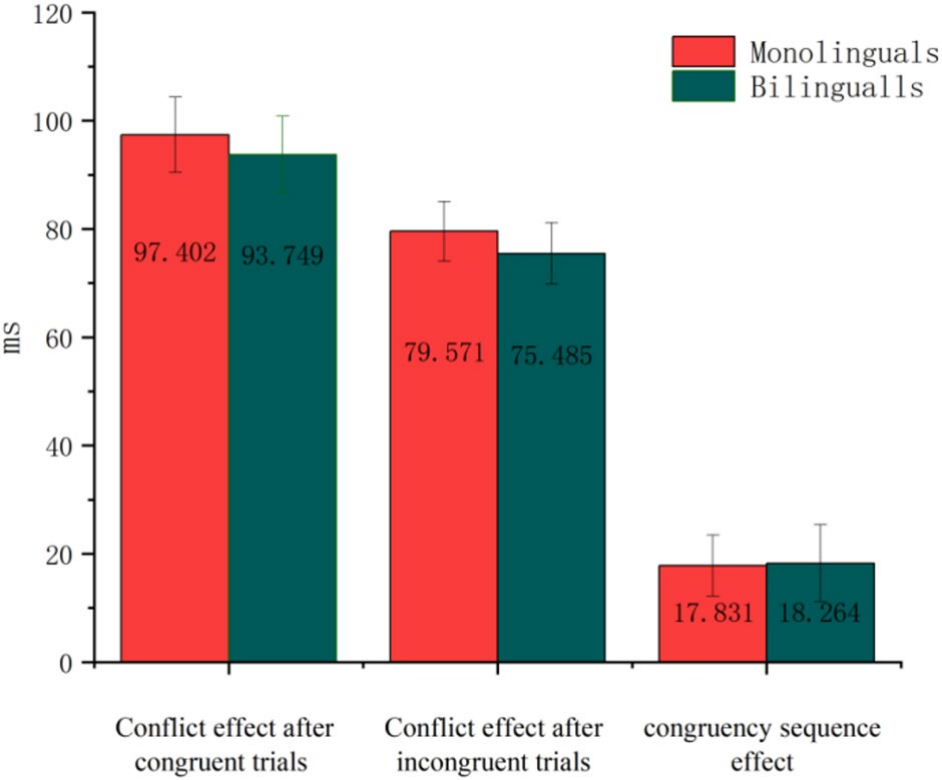
Conflict effect and congruency sequence effect results.

An independent samples t-test was conducted to compare the congruency sequence effect (CSE = (cI − cC) − (iI − iC)) on response times between monolingual and bilingual participants. Results showed no significant difference in CSE magnitude between the groups, *t*(43) =  − 0.048, *p* = 0.962. The results for both conflict effects and the congruency sequence effect are illustrated in Fig. 3.

### Electroencephalography-N2 component

#### Average amplitude analysis

A four-way repeated measures ANOVA with factors of Group (2: monolingual, bilingual), Previous Trial Consistency (2: congruent [c], incongruent [i]), Current Trial Consistency (2: congruent [c], incongruent [i]), and Brain Region (3: left, centre, right) was conducted on the mean amplitude of the N2 component. Previous trial consistency, current trial consistency, and brain region were within-subject variables, and group was a between-subject variable.

Results revealed a significant main effect of Group, *F*(1, 43) = 9.278, *p* = 0.004, η_p_^2^ = 0.177, with the monolingual group exhibiting significantly larger N2 amplitudes than the bilingual group. The main effect of Current Trial Consistency was also significant, *F*(1, 43) = 4.779, *p* = 0.034, η_p_^2^ = 0.100, showing greater N2 amplitude in the current trial incongruent condition compared to the congruent condition. Additionally, there was a significant main effect of Brain Region, *F*(2, 86) = 5.442, *p* = 0.006, η_p_^2^ = 0.112, with significantly larger amplitudes observed in the central region than in the right hemisphere (*p* = 0.003), as shown in Fig. 7a.

A significant interaction between Previous Trial Consistency and Current Trial Consistency was found, *F*(1, 43) = 4.440, *p* = 0.041, ηp2 = 0.094. Simple effects analyses indicated that when the previous trial was congruent, the N2 amplitude was significantly larger in the current trial incongruent condition (cI) compared to the congruent condition (cC) (*p* = 0.005), i.e., cI > cC. However, when the previous trial was incongruent, no significant difference was found between current incongruent (iI) and congruent (iC) conditions (*p* = 0.390). In the current incongruent condition, the N2 amplitude was significantly greater following a previous congruent trial (cI) than following a previous incongruent trial (iI) (*p* = 0.027), i.e., cI > iI. Conversely, in the current congruent condition, the difference between previous congruent (cC) and previous incongruent (iC) trials was not significant (*p* = 0.471).

**Fig. 4:**
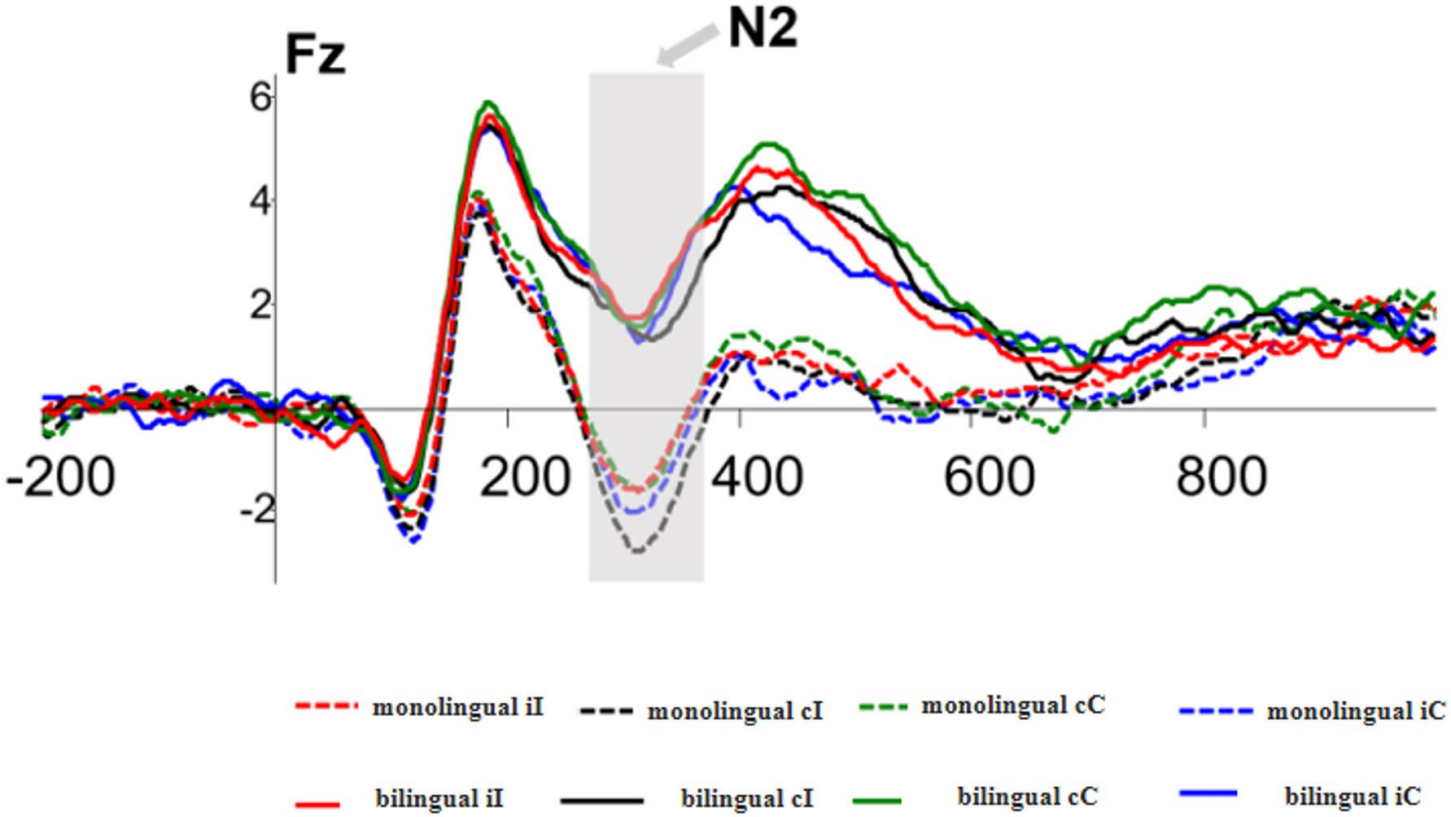
Average wave amplitude represented by Fz.

All other main effects and interactions were nonsignificant (*F*s < 2.6). See Fig. 4 for N2 amplitude results and Fig. 7a for topographical maps.

#### Conflict effects size analysis

**Fig. 5:**
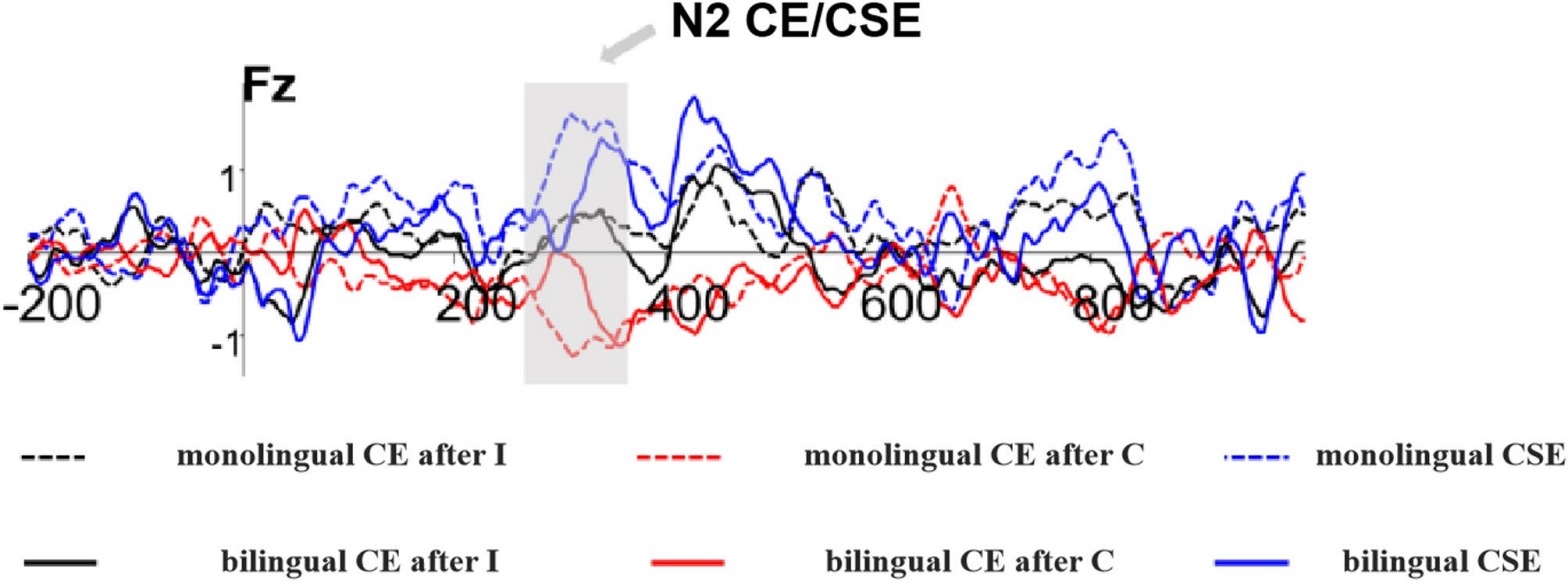
Conflict effect and congruency sequence effect represented by Fz.

A 2 (Group: monolingual, bilingual) × 2 (Conflict Effect Type: post-consistent trial, post-inconsistent trial) × 3 (Brain Region: left, centre, right) repeated measures ANOVA was conducted on the magnitude of conflict effects in the N2 component amplitudes. Results showed a significant main effect of Conflict Effect Type, *F*(1, 43) = 4.440, *p* = 0.041, η_p_^2^ = 0.094, with larger conflict effects following consistent trials than inconsistent trials. No significant main effects of Group or Brain Region, nor any significant interactions, were found (all *F*s < 2.6). The conflict effect sizes for the N2 component are illustrated in Fig. 5.

#### Congruency sequence effect size analysis

A 2 (Group: monolingual, bilingual) × 3 (Brain Region: left, centre, right) repeated measures ANOVA was performed on the congruency sequence effect (CSE) magnitude in the N2 component amplitude. Neither the main effects of Group and Brain Region nor their interaction were significant (all *F*s < 2.6). The magnitude of the CSE on the N2 component is displayed in Fig. 5, with a corresponding topographical map shown in Fig. 9a.

### Electroencephalography-P3 component

#### Average amplitude analysis

**Fig. 6:**
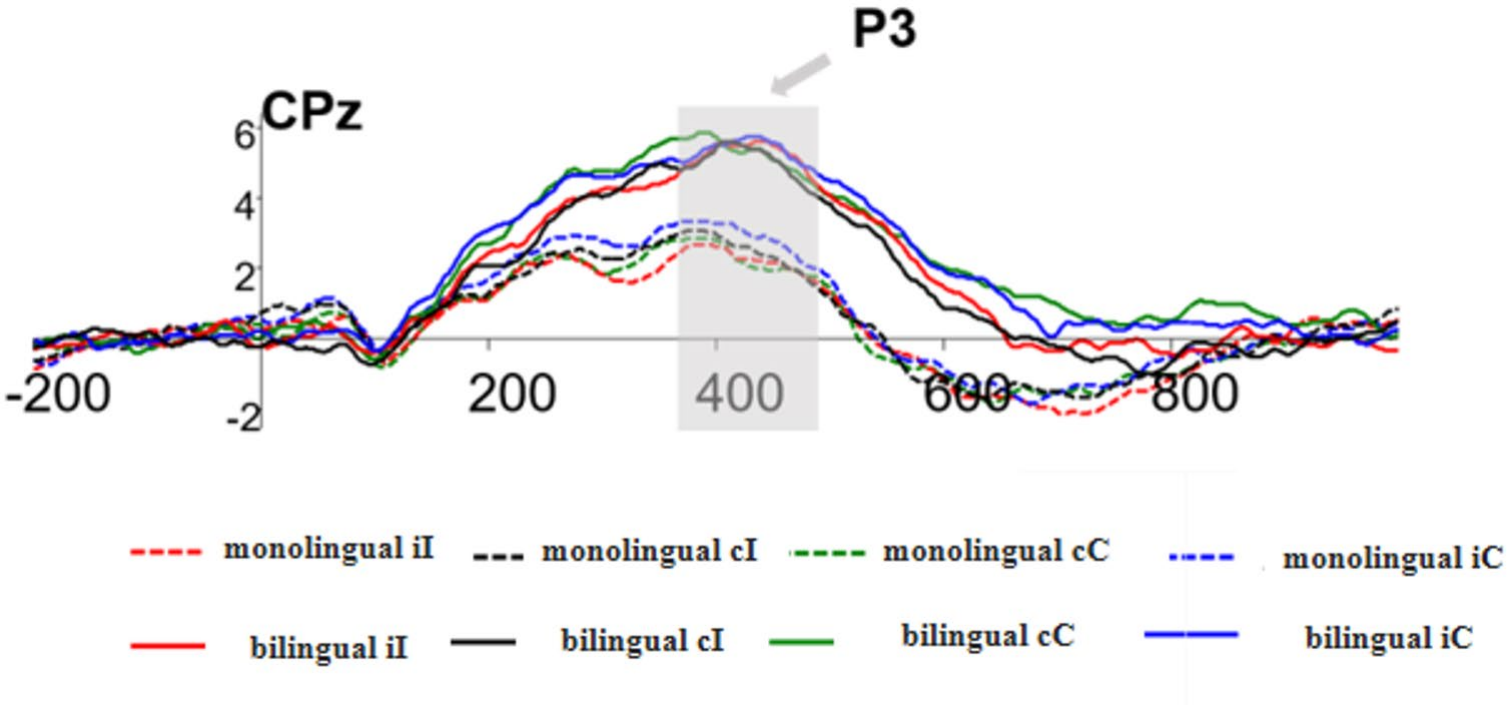
Average wave amplitude represented by CPz.

**Fig. 7:**
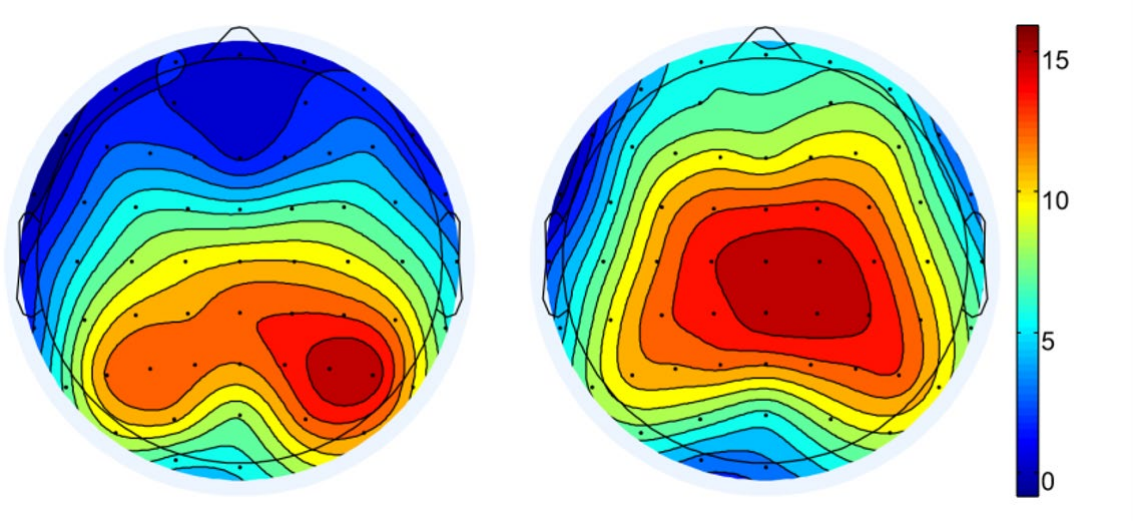
**a** N2 topographic map. **b** P3 topographic map.

A four-factor repeated measures ANOVA with 2 (Group: monolingual, bilingual) × 2 (Previous Trial Consistency: c, i) × 2 (Current Trial Consistency: C, I) × 3 (Brain Region: left, centre, right) was conducted, using the mean amplitude of the P3 component as the dependent variable. The results revealed a significant main effect of Group, *F*(1, 43) = 8.570, *p* = 0.005, η_p_^2^ = 0.166, with the monolingual group showing significantly smaller P3 amplitudes than the bilingual group. The main effect of Brain Region was also significant, *F*(2, 86) = 10.346, *p* < 0.001, η_p_^2^ = 0.194, with amplitudes in the central region significantly larger than those in both the left (*p* < 0.001) and right (*p* = 0.038) regions. No significant difference was found between the left and right regions. All other main effects and interactions were nonsignificant (*F*s < 2.4). See Fig. 6 for P3 amplitudes and Fig. 7b for the topographical map.

#### Conflict effect size analysis

**Fig. 8:**
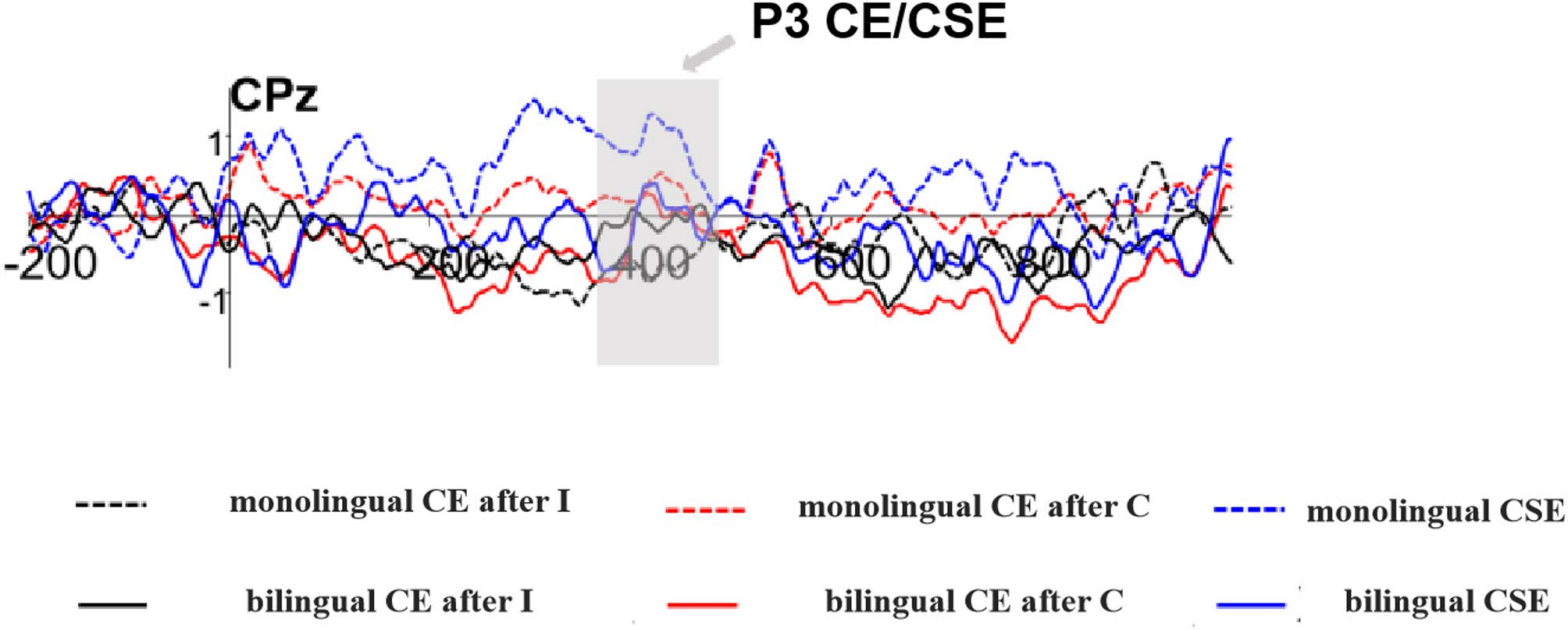
Conflict effects and congruency sequence effect represented by Fz.

A repeated-measures ANOVA with 2 (Group: monolingual, bilingual) × 2 (Conflict Effect Type: post-consistent trial, post-inconsistent trial) × 3 (Brain Region: left, centre, right) was conducted on the amplitude of the P3 component. The analysis revealed no significant main effects or interactions, except for a significant interaction between Conflict Effect Type and Brain Region, *F*(2, 86) = 7.434, *p* = 0.001, η_p_^2^ = 0.147. Simple effects analyses indicated that after inconsistent trials, the conflict effect was significantly greater in the right brain region than in the left (*p* = 0.040). After consistent trials, the conflict effect was slightly greater in the left brain region than in the right, with a borderline significant difference (*p* = 0.053). The conflict effect amplitudes for the P3 component are shown in Fig. 8.

#### Congruency sequence effect (CSE) size analysis

**Fig. 9:**
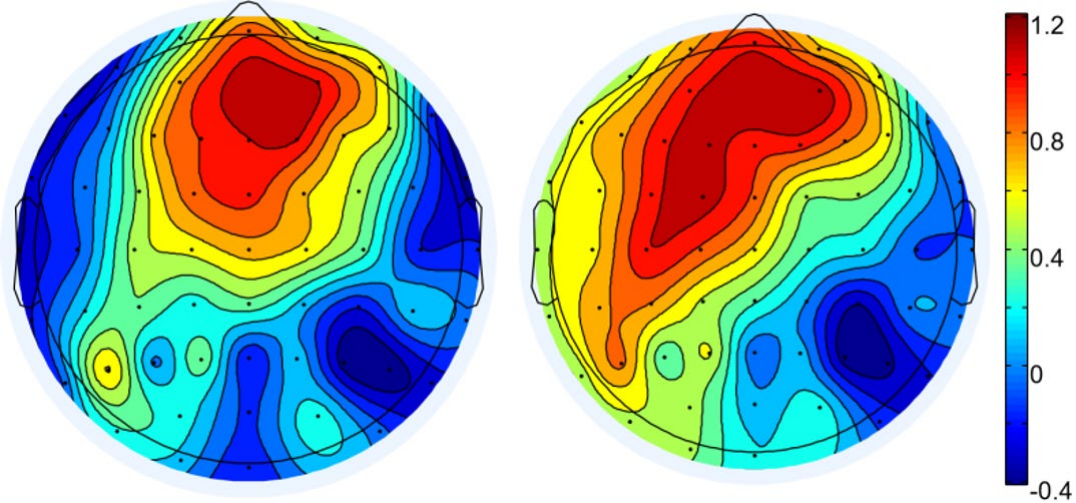
**a** N2 CSE topographic map. **b** P3 CSE topographic map.

A repeated-measures ANOVA with 2 (Group: monolingual, bilingual) × 3 (Brain Region: left, centre, right) was conducted on the amplitude of the P3 component CSE. The analysis showed a significant main effect of Brain Region, *F*(2, 86) = 7.435, *p* = 0.001, η_p_^2^ = 0.147. Post hoc comparisons revealed that the CSE amplitude was significantly larger in the left brain region than in the right (*p* = 0.001), whereas the central region did not significantly differ from either the left or right regions. The main effect of Group was not significant, *F*(1, 43) = 1.825, *p* = 0.184, nor was the Group × Brain Region interaction, *F*(2, 86) = 0.473, *p* = 0.625. The CSE amplitude results for the P3 component are displayed in Fig. 8, with topographical maps shown in Fig. 9b.

## Discussion

The present study investigated whether bilingual experience modulates cognitive control processes in a Flanker task, with a particular focus on two key processes: conflict monitoring, which reflects the detection and adjustment to conflicting information, and suppression control, which reflects the ability to inhibit competing responses. Conflict monitoring was indexed by overall response times/amplitudes and the congruency sequence effect (CSE), whereas suppression control was indexed by the conflict effect (CE). At the behavioral level, no significant group differences in overall response times were observed between monolinguals and bilinguals. In contrast, ERP analyses revealed group differences in both the N2 and P3 components, suggesting that monolingual and bilingual participants engage partially distinct neural mechanisms when resolving conflict. Thus, although behavioral performance appeared similar across groups, the underlying neural dynamics of conflict monitoring and suppression control diverged. The following sections elaborate on these findings, with the discussion organized around conflict monitoring, suppression control and differences in brain regions.

### Conflict monitoring-overall response time/amplitude

Overall reaction time is a traditional behavioral indicator of conflict monitoring. In the present study, no significant differences in overall reaction time were found between bilinguals and monolinguals, suggesting that bilinguals did not exhibit an advantage in conflict monitoring at the behavioral level. This result aligns with previous findings^34^. Some researchers have noted that the influence of bilingual experience on conflict monitoring may not be readily observable through behavioral outcomes alone, and that neurophysiological indicators may offer deeper insights into the relationship between bilingualism and cognitive control^35^.

Indeed, our ERP findings revealed between-group differences in both the N2 and P3 components. Specifically, bilinguals exhibited smaller N2 amplitudes than monolinguals under both congruent and incongruent conditions. This finding is consistent with results reported by Kousaie et al. ^14^. The N2 component, an event-related potential associated with conflict monitoring and cognitive control, typically reflects the extent of conflict detection, with greater N2 amplitudes indicating greater engagement of monitoring processes^36^. Thus, the smaller N2 amplitudes observed in bilinguals may reflect more efficient language processing and better regulation of cognitive resources, especially in switching between languages, resulting in reduced cognitive load during task performance.

This interpretation is supported by Lamm et al., who found that N2 amplitude decreases from childhood to adolescence, reflecting the maturation of cognitive control^37^. Smaller N2 amplitudes have been associated with better performance on independent executive function tasks. Additionally, bilinguals have been shown to exhibit reduced activation in the anterior cingulate cortex (ACC) compared to monolinguals, which corresponds to lower N2 amplitudes and suggests more efficient ACC engagement^38^. Yeung and Cohen also noted that increased N2 amplitude may reflect enhanced processing of task-irrelevant information^23^. Collectively, these findings support the interpretation that bilinguals’ reduced N2 amplitudes reflect more efficient conflict monitoring mechanisms at the neural level.

Regarding the P3 component, the mean amplitude across the two groups exhibited a pattern opposite to that observed for the N2 component: bilinguals showed significantly larger P3 amplitudes than monolinguals. The P3 is considered a reliable neural marker of attentional resource allocation, with greater amplitudes reflecting increased attention engagement^26^. Thus, the enhanced P3 amplitudes observed in bilinguals suggest that they recruit more cognitive resources when performing cognitive tasks.

This finding is consistent with previous research. For instance, Kousaie et al., using Simon, Stroop, and Flanker tasks, also reported that bilinguals exhibited greater P3 amplitudes than monolinguals, indicating heightened attentional engagement across a range of conflict monitoring paradigms^39^.

Although bilinguals and monolinguals performed similarly at the behavioral level, neurophysiological data suggest distinct underlying mechanisms. Bilinguals exhibited reduced N2 amplitudes-possibly reflecting more efficient conflict monitoring and reduced need for anterior cingulate cortex (ACC) engagement-yet showed increased P3 amplitudes, indicating greater attentional allocation. Together, these findings imply that bilinguals may be more efficient in resolving conflict and mobilizing attentional resources.

Importantly, these results also suggest that Dai-Chinese bilinguals demonstrate similar cognitive advantages and underlying mechanisms as bilinguals who have varying other second languages.This supports the view that the cognitive benefits of bilingualism are not restricted to specific language pairs or cultural contexts, but rather represent a universal phenomenon.

### Conflict monitoring- congruency sequence effects

The congruency sequence effect (CSE) is another important index of conflict monitoring. In the present study, analysis of conflict effect size revealed that the magnitude of conflict effects was greater following congruent trials than after incongruent trials. This behavioral pattern reflects a serial consistency effect, commonly referred to as the conflict adaptation effect. It suggests that individuals dynamically adjust their attentional strategies based on the congruency of the previous trial. Specifically, when the preceding trial is incongruent, individuals are more likely to engage in conflict monitoring, which reduces the conflict effect on the subsequent trial.

The conflict adaptation effect reflects experience-driven modulation of attention in response to cognitive conflict. In EEG results, this pattern was observed on the N2 component: the conflict effect was significantly larger following congruent than incongruent trials, indicating that the neural mechanism for conflict monitoring is sensitive to sequential congruency. However, this pattern was not observed on the P3 component, where the conflict effect did not differ significantly between post-congruent and post-incongruent trials. This suggests that while conflict adaptation affects monitoring processes (as indexed by the N2), it may not influence attentional resource allocation (as indexed by the P3).

The CSE is thought to reflect an individual’s ability to adaptively regulate attention and cognitive control in response to previous conflict. Grundy et al. reported that bilinguals exhibited a smaller conflict adaptation effect than monolinguals, despite no group differences in overall reaction times^21^. They interpreted this as evidence that bilinguals are better at dissociating their attentional responses to congruent versus incongruent stimuli. However, the present study found no significant group differences in CSE magnitude, either in behavioral measures or in EEG components. This aligns with previous research by Goldsmith and Morton, who also found no differences between bilingual and monolingual adults using the Flanker task^40^. Similarly, studies using the size congruency task, Simon task, and Attention Network Task (ANT) have reported no significant group differences in conflict adaptation effects^28,41^.

Taken together, our results suggest that bilinguals do not show an advantage over monolinguals in terms of conflict adaptation or in the dynamic adjustment of cognitive resources. Although bilinguals demonstrated a monitoring advantage-as evidenced by differences in N2 and P3 amplitudes-this advantage appears limited to the conflict detection process itself and does not extend to the adaptive modulation of conflict monitoring.

### Suppression control

The conflict effect refers to the negative influence of conflicting information on task performance during cognitive processing. Behaviorally, it manifests as increased reaction times in incongruent conditions compared to congruent ones. In the present study, participants responded significantly faster in congruent trials than in incongruent trials, indicating the presence of a classic conflict effect.

When considering the congruency relationship between the preceding and current trials, response times in the cC condition (congruent preceded by congruent) were significantly shorter than in the cI condition (incongruent preceded by congruent), and response times in the iC condition (congruent preceded by incongruent) were also shorter than those in the iI condition (incongruent preceded by incongruent). These results indicate that conflict effects were observed after both congruent and incongruent trials, demonstrating the stability of this effect.

Consistent with the behavioral findings, the EEG results showed that N2 amplitudes were significantly larger in incongruent trials compared to congruent trials, suggesting the presence of conflict effects at the neural level as well. Specifically, when the preceding trial was congruent, a significant conflict effect was observed on the N2 component of the current trial. When the preceding trial was incongruent, although the difference did not reach statistical significance, the N2 amplitude in the incongruent condition was still numerically larger than in the congruent condition, reflecting a trend consistent with conflict processing.

Conflict effect size-defined as the difference in mean reaction times or EEG amplitudes between incongruent and congruent trials-is commonly used as an index of inhibitory control. A smaller conflict effect size typically indicates stronger inhibitory control. Some studies have reported that bilinguals exhibit smaller conflict effects, suggesting a greater ability to inhibit irrelevant or conflicting information compared to monolinguals^3,42,43^.

However, the present study found no significant differences in conflict effect size between bilinguals and monolinguals in behavioral outcomes. This finding is consistent with previous studies, all of which reported no significant association between bilingual experience and conflict effect sizes in reaction time measures using the Flanker or Stroop tasks^19,21,44^. Similarly, EEG results showed no significant differences between groups in conflict effect size on either the N2 or P3 components. These converging findings suggest that, contrary to some prior claims, bilinguals may not exhibit a general advantage in inhibitory control as measured by conflict effect size.

### Differences in brain regions

For right-handed individuals, the dominant hemisphere is typically the left hemisphere of the brain, which is also heavily involved in language functions^45,46^. Unlike monolinguals, bilinguals have experience managing two languages, which may engage more language-related control regions in the left hemisphere, such as the left caudate nucleus and the left inferior frontal gyrus (LIFG) ^47^. Structural studies have also reported that bilinguals show greater cortical thickness in the LIFG ^48^ and the left middle frontal gyrus ^41^, supporting the view that bilingual advantages could be linked to left-hemisphere regions.

However, in the present study, no significant group differences (monolinguals vs. bilinguals) were observed in any of the EEG components of interest-including waveform amplitudes, conflict effects, or congruency sequence effects (CSE)-with respect to brain region activation. This indicates that bilinguals did not exhibit greater activation in left hemisphere regions compared to monolinguals.

At the same time, the analyses revealed that both the N2 and P3 amplitudes were generally largest at midline electrodes, suggesting that conflict processing relies on bilateral rather than strongly lateralized mechanisms. An additional finding was that, for the P3 component, the magnitude of the CSE effect was larger in the left hemisphere than the right, and after incongruent trials the conflict effect appeared greater on the right. Importantly, these hemispheric asymmetries were observed across participants regardless of language group, and therefore should not be taken as evidence of a bilingual-specific left-hemisphere advantage. Instead, they may reflect general lateralization patterns in attentional control and conflict adaptation. Future studies using more fine-grained neuroimaging techniques are needed to clarify the functional significance of these asymmetries.

## Limitations and future directions

This study has several limitations that point to potential directions for future research.

First, while we considered participants’ language proficiency, we did not collect detailed information on language usage or language switching frequency. Recent research has increasingly highlighted the value of moving beyond dichotomous measures of bilingualism ^49^, suggesting that more fine-grained assessments-such as the relative use of each language, switching frequency, and context-specific engagement-may offer richer insights into the ways bilingual experience relates to cognitive control. In addition, the Dai-Chinese bilingual participants in the present study exhibited higher proficiency in Chinese than in their ethnic language. This pattern reflects the sociolinguistic context of ethnic minority regions in China, where Chinese is the dominant language of education and daily communication. However, this feature also represents a limitation of the current study, as our bilingual sample may not fully capture the diversity of bilingual experiences. Future research should include bilinguals with more balanced or different proficiency profiles to provide a more comprehensive understanding of how language experience influences cognitive control.

Second, the present study assessed ERP effects using mean amplitude measures within predefined time windows, without analyzing peak amplitude or latency. Although mean amplitude is generally considered more robust and reliable, particularly in the presence of noise, latency variability, and component overlap ^50–52^, peak amplitude and latency can provide complementary information about the magnitude and timing of cognitive processes. Future research may benefit from combining these approaches to achieve a more comprehensive understanding of bilingualism-related differences in cognitive control mechanisms.

Third, the apparent hemispheric differences observed in the P3 component represent another limitation. In the present study, the CSE appeared relatively larger in the left hemisphere, whereas the CE was more pronounced in the right hemisphere. While these patterns might hint at potential hemispheric differences in conflict adaptation and suppression control, the limited spatial resolution of ERP makes it difficult to precisely attribute these effects to specific neural sources^48^.

Fourth, the hemispheric differences observed in the P3 component also represent a limitation. In the present study, the CSE appeared relatively larger in the left hemisphere, whereas the CE was more pronounced in the right hemisphere. While these patterns may suggest potential hemispheric differences in conflict adaptation and inhibitory control, the limited spatial resolution of ERP makes it difficult to precisely attribute these effects to specific neural sources^48^. Moreover, in our design, the number of electrodes representing each hemisphere was not fully balanced (left: 3, midline: 1, right: 3), which may have given disproportionate weight to the midline electrode. Although midline sites such as Fz and CPz are widely recognized in the literature as representative of frontal and centro-parietal midline activity^48,49^, this methodological choice could still affect hemispheric comparisons. Importantly, existing evidence for hemispheric asymmetries in P3 amplitude during conflict tasks remains scarce and inconsistent, and thus these observations should be interpreted with caution. Future research could address this limitation by using an equal number of electrodes per hemisphere or by combining ERP with source localization techniques for greater precision.

Finally, our sample consisted exclusively of young adult right-handed participants, which limits the generalizability of the findings. Future studies should consider more diverse age ranges, handedness, and language backgrounds to determine the robustness of bilingual effects on cognitive control.

## Conclusions

This study used the Flanker task and ERP techniques to compare cognitive control between Dai–Han bilinguals and monolinguals, aiming to clarify which cognitive mechanisms might underlie any bilingual advantage. No significant group differences were observed in behavioral reaction times, Conflict Effects (CE), or Congruency Sequence Effects (CSE). In contrast, ERP analyses revealed smaller N2 amplitudes and larger P3 amplitudes in bilinguals relative to monolinguals, suggesting differences in the neural correlates of conflict monitoring even in the absence of behavioral differences. No clear left-hemisphere dominance was observed; instead, both groups showed bilateral activation, though the left hemisphere exhibited a stronger CSE in P3 amplitude. This may imply lateralized attentional adaptation, warranting further research. Overall, the results provide partial support for a potential bilingual advantage in conflict monitoring at the neural level, as reflected in N2 and P3 amplitudes, but do not support an advantage in inhibitory control (CE). These results, based on Dai–Han bilinguals, contribute to understanding bilingual cognitive control in non-Indo-European language contexts.

## Data Availability

The datasets used and/or analyzed during the current study are available from the corresponding author on reasonable request.
